# Lead (Pb) Accumulation in Human THP-1 Monocytes/Macrophages In Vitro and the Influence on Cell Apoptosis

**DOI:** 10.1007/s12011-020-02215-7

**Published:** 2020-06-15

**Authors:** Emilia Metryka, Patrycja Kupnicka, Patrycja Kapczuk, Beata Aszakiewicz, Katarzyna Piotrowska, Marta Tkacz, Izabela Gutowska, Dariusz Chlubek, Irena Baranowska-Bosiacka

**Affiliations:** 1 Department of Biochemistry and Medical Chemistry, Pomeranian Medical University in Szczecin, Powstańców Wlkp. 72, 70-111 Szczecin, Poland; 2 Department of Physiology, Pomeranian Medical University in Szczecin, Powstańców Wlkp. 72, 70-111 Szczecin, Poland; 3 Department of Medical Chemistry, Pomeranian Medical University in Szczecin, Powstańców Wlkp. 72, 70-111 Szczecin, Poland

**Keywords:** Lead (Pb), Apoptosis, Pb accumulation, Neurodegeneration, THP-1 cells

## Abstract

In this study, we investigated the ability of THP-1 monocytes and macrophages to accumulate lead (Pb) in vitro, relative to Pb concentration and length of exposure. Moreover, we also evaluated the effect of Pb accumulation on cell viability and apoptosis. THP-1 monocytes and macrophages were cultured in the presence of Pb at 1.25 μg/dL, 2.5 μg/dL, 5 μg/dL, and 10 μg/dL. Pb accumulation was examined by inductively coupled plasma and confocal microscopy. The influence of Pb on cell viability, apoptosis, and necrosis was assessed using flow cytometry. The results showed that Pb was toxic to THP-1 monocytes/macrophages even at very low environmental concentrations. Despite the use of low concentrations, both monocytes and macrophages showed dose-dependent and time-dependent decreases in viability, with a simultaneous increase in the percentage of early and late apoptotic cells. Macrophages reacted more strongly to Pb than monocytes. When exposed to the same Pb concentrations, they showed lower viability and a higher percentage of necrotic cells. The incubation time positively correlated with Pb accumulation in a dose-dependent manner. The obtained results indicate that environmental exposure to low Pb concentrations may significantly impair the function of macrophages, with the increased number of apoptotic cells potentially contributing to the development of many pathologies in the brain and whole body.

## Introduction

Despite numerous prevention efforts in recent years [1–4], lead (Pb) is still considered to be one of the main substances with the greatest potential concern for human health. It was classified in 2017 as a priority list 2 substance by the Agency for Toxic Substances and Disease Registry [5]. Although acute Pb poisoning is currently rare [6, 7], chronic human exposure to low Pb concentrations remains a public health concern, especially in large agglomerations and industrial areas [8–10].

The brain is particularly sensitive to the neurodegenerative and neuropathic effects of Pb [11, 12], with even low levels of Pb during body development resulting in cognitive disorders [13–19]. Later in life, Pb accelerates the progress and symptoms of age-related neurodegenerative diseases such as Alzheimer’s disease and Parkinson’s disease [20–22]. The immune system also seems to be one of the more sensitive targets for Pb. Although at low environmental concentrations, Pb is not able to cause overt damage to the main immune cells and does not result in deficiencies in immune cells that are determined by routine tests, it does adversely affect the regulation and function of immune cells [23]. Pb can also act as a strong pro-inflammatory factor in the brain [12] and in the whole body [11].

At the cellular level, Pb disrupts the energy state of cells, causing ultrastructural and functional disorders in mitochondrial metabolism by decreasing mitochondrial membrane potential, depleting adenosine triphosphate (ATP) pool, and increasing the production of reactive oxygen species [24]. Moreover, Pb influences the expression of mRNAs in the immediate early genes fos and jun [25–27], inhibits DNA repair, and exerts indirect genotoxic effects by acting as a co-mutagen [28]. Our previous data also shows that exposure to Pb in rats decreases the number of hippocampal neurons without severe apoptosis or necrosis [29], although a number of studies suggest that Pb does induce apoptosis in several cell types [29–34]. Excessive or disturbed apoptosis is mentioned as a major factor in the formation and propagation of autoimmune, neuropathic, and neurodegenerative diseases induced by Pb exposure [12, 35, 36].

Macrophages play a key role in the development of the aforementioned disorders induced by Pb. This very diverse group, with different functions in different organs [37], includes microglia in the central nervous system [38, 39]. Through their ability to phagocytose, they protect the organism from a wide array of xenobiotics [40, 41], so macrophages constitute a good experimental model to study Pb-induced inflammation of brain tissues [42] to provide a simplified/approximate experimental model to study the effects of Pb exposure in microglia.

In a previous study, we presented the direct effect of Pb on neurodegeneration in the brain, particularly the expression of selected proteins, the activity of enzymes, and the expression of receptors participating in neurodegeneration processes [21, 22, 43]. The aim of this present study was to evaluate Pb accumulation in THP-1 (Tohoku Hospital Pediatrics-1) macrophages after exposure to low Pb concentrations (reflecting chronic environmental exposure to this metal), and the effect of Pb accumulation on apoptosis in these cells. The Pb concentrations and exposure times used in this study have not yet been studied in terms of their effect on apoptosis. Because even small changes in apoptosis induced by environmental factors (such as Pb) might influence genetic instability [44], the results of our study may help to better understand the mechanisms of Pb toxicity.

## Materials and Methods

### Reagents

THP-1 cells came from the American Type Culture Collection (ATCC, Rockville, USA), while RPMI 1640 culture medium and phosphate-buffered saline (PBS) from Biomed Lublin (BIOMED-LUBIN, Poland). Antibiotics (penicillin and streptomycin) were purchased from Sigma-Aldrich (Poznan, Poland) while fetal bovine serum (FBS) from Gibco (Paisley, UK). Lead acetate (PbAc) used for the preparation of solutions came from Sigma-Aldrich (Poznan, Poland). Phorbol myristate acetate (PMA) required for the transformation of monocytes into macrophages was purchased from Sigma-Aldrich (Poznań, Poland). Annexin V/fluorescein isothiocyanate (FITC) apoptosis evaluation kit came from BD Pharmingen (USA). Nitric acid (V) (HNO_3_) (Suprapur, Merck, Germany) and Triton (Triton X-100, Sigma) were used for medium digestion in a microwave oven. Inductively coupled plasma (ICP) IV multi-element standard solution (Merck) was used to form a calibration curve. These solutions were made using deionized water (Direct Q UV, Millipore, approx. 18.0 MΩ). The penetration of Pb ions into the cells was examined using Leadmium™ Green AM (molecular probes). Formalin from Sigma-Aldrich (Poznań, Poland) was used to fix the cells on a microscope slide.

### Cell Culture and Treatment

RPMI 1640 medium supplemented with 10% FBS, 100 IU/mL penicillin, and 10 μg/mL streptomycin at 37 °C, 5% CO_2_, and 95% humidity were used to culture the cells. The cells were passaged three times a week to maintain a density below 8 × 10^5^ cells/mL. During the experiment, the cells were incubated with 100 nM PMA for 24 h to differentiate monocytes in macrophages. After three washes with warm PBS, macrophages added to the medium were incubated for 48 h with lead acetate (PbAc) at four concentrations: 1.25 μg/dL, 2.5 μg/dL, 5 μg/dL, 10 μg/dL. The control sample was cultured without the addition of irritants. After the incubation time, the cells were removed from the medium with trypsin and transferred to tubes. Centrifugation (125×*g* for 6 min) was used to obtain cell pellets used in the next stages of the research.

### THP-1 Macrophages Experimental Model

This study used THP-1 leukemia cells (acute monocytic leukemia, FAB type M5), the most popular line in research on inflammatory response mechanisms for the last 30 years, i.e., since the isolation of this cell line [45]. THP-1 leukemia cells are also frequently used in various studies on the physiology or pathology of human monocytes and macrophages [42, 46–50].

PbAc solution with levels of 1.25 μg/dL, 2.5 μg/dL, 5 μg/dL, and 10 μg/dL Pb were used in in vitro cultures of THP-1 monocytes and macrophages. Our previous studies [8] indicate that the former two levels are in the range found in the whole blood and cord blood of young women living in northern Poland and their newborn children. The Pb blood level of 5 μg/dL is the threshold PbB concentration for children and pregnant women, while 10 μg/dL is the threshold PbB level for adults [51].

### Flow Cytometry Measurement of Cell Viability

Flow cytometry using Annexin V-FITC staining in combination with iodide propidium (PI) was used to determine THP-1 cell viability. FITC Annexin V and PI identified live cells as negative. Early apoptotic cells were FITC Annexin V positive and PI negative. Late apoptotic/necrotic cells were FITC Annexin V positive and PI positive. A Navios (Beckman Coulter, USA) flow cytometer was used to carry out the tests.

### Quantitative Evaluation of Apoptosis by Flow Cytometry

An Annex V/propidium iodide test was used to identify apoptotic and necrotic cells. FITC-conjugated Annex V protein (fluorescein isothiocyanate) was used to identify the externalizations of phosphatidylserine as an early apoptosis marker. Cell membrane damage was detected by binding PI (propidium iodide) to nuclear DNA. A Navios flow cytometer (Beckman Coulter, USA) was used to analyze the cells. Apoptosis was determined using an apoptosis detection kit according to the manufacturer’s instructions.

### Lead Ion Accumulation in Cells Assessed by ICP-OES

The levels of Pb in THP-1 macrophages and culture medium were determined following 24- and 48-h exposure to the tested Pb concentrations, with the use of atomic emission spectrometry with excitation in inductively coupled plasma (ICP-OES, ICAP 7400 Duo, Thermo Scientific). A MARS 5, CEM system was used to carry out the microwave digestion of the samples. The samples were first transferred to pure polypropylene tubes, to which 1 mL of 65% HNO_3_ and 1 mL of non-stabilized 30% hydrogen peroxide (H_2_O_2_) were added. The samples were then transferred to Teflon vessels and placed in a microwave digestion oven. First, the samples were gradually heated to 180 °C within 15 min. Then, the temperature was maintained at 180 °C for the next 20 min. Then, the samples were transferred to pure polypropylene tubes. The products of digestion were diluted 20 times. Then, an internal standard yttrium (final concentration in the 0.5-mg/L sample) and 1 mL 1% Triton were added to a 500-μL sample. 0.075% HNO_3_ was used to supplement the samples to a final volume of 10 mL and stored in a refrigerator (4–8 °C) until analysis. A blank sample was prepared according to the same scheme in which 250 μL of nitric acid (*V*) was added instead of the test sample. Multi-elemental standard solutions were used to prepare the calibration curve. Deionized water was used to prepare all solutions (~ 18.0 MΩ). Analysis was based on the wavelengths of 220 nm and 353 nm.

### Visualization of Lead Ion Accumulation in Cells Evaluated by Confocal Microscopy

Confocal microscopy was used to examine lead ion accumulation in THP-1 macrophages. Macrophages were cultured on glass slides for 48 h in a complete medium with lead acetate at the aforementioned concentrations. Following the completion of cell incubation, the slides were washed with PBS and fixed with 4% buffered formalin for 15 min at room temperature. After fixation and washing with PBS, the cells were permeabilized with 0.5% Triton X-100 solution in PBS. The penetration of Pb ions into the cells was evaluated by adding Leadmium™ Green AM dye. Cells were examined under a confocal microscope (FV1000) with an inverted IX81 microscope (Olympus, Germany). The best signal resolution from Hoechst 33258 and FITC fluorescence was obtained thanks to the use of three-channel acquisition and sequential scanning. Fluorescent images were combined with transient light images.

### Statistical Analysis

Statistica 10.0 software was used to analyze the obtained results, using arithmetical mean ± SD for each of the studied parameters. The Shapiro-Wilk *W* test was used to determine the distribution of results for individual variables. Non-parametric tests were used for further analyses since most of the distributions were not normal. The differences between the groups studied were assessed using the non-parametric Mann-Whitney *U* test. Differences were deemed statistically significant when *p* ≤ 0.05.

## Results

### Lead and Cell Viability

**Fig. 1:**
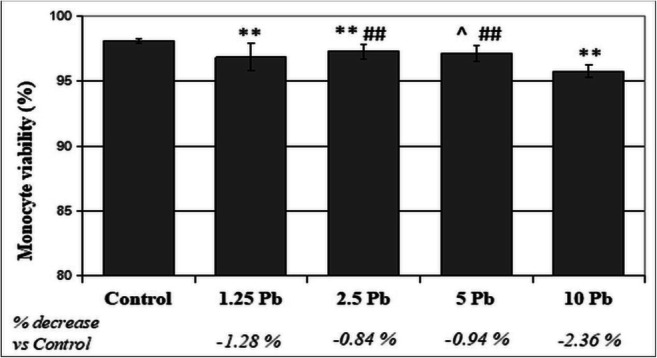
The effect of Pb on the vitality of THP-1 monocytes cultured with various lead acetate solutions for 48 h. After incubation cell viability was measured by flow cytometry analysis (*n* = 6). **Statistically significant difference to control (*p* ≤ 0.005). ##Statistically significant difference to 10 μg/dL PbAc (*p* ≤ 0.005). ^Statistically significant difference to 1.25 μg/dL PbAc (*p* ≤ 0.05). Control—cells incubated in RPMI medium with 10% FBS and without Pb

**Fig. 2:**
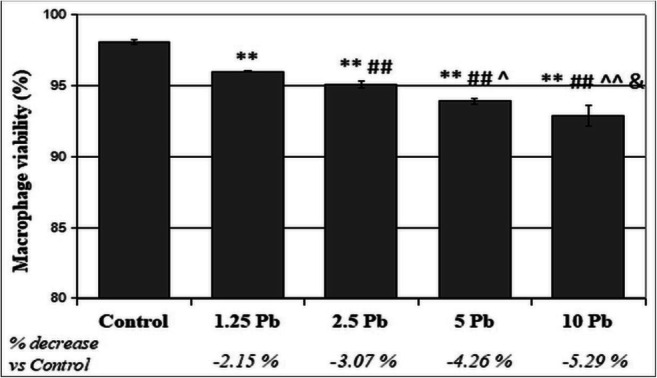
The effect of lead on the viability of THP-1 macrophages cultured with various lead acetate solutions for 48 h. After incubation, cell viability was measured by flow cytometry analysis (*n* = 6). **Statistically significant differences in comparison with the control (*p* ≤ 0.005). ##Statistically significant differences in comparison with 1.25 μg/dL Pb (*p* ≤ 0.005). ^^Statistically significant differences in comparison with 2.5 μg/dL Pb (*p* ≤ 0.005), ^(*p* ≤ 0.05). Statistically significant differences in comparison with 5 μg/dL Pb (*p* ≤ 0.05). Control—cells incubated in RPMI medium with 10% FBS and without Pb

THP-1 monocytes and macrophages were cultured for 48 h with exposure to different PbAc concentrations: 1.25 μg/dL, 2.5 μg/dL, 5 μg/dL, 10 μg/dL. The viability of both monocytes and macrophages decreased with an increase in the applied Pb concentration (Figs. 1 and 2).

The THP-1 evaluation of monocytes showed a statistically significant decrease in the viability of cells exposed to Pb at 1.25 μg/dL, 2.5 μg/dL, and 10 μg/dL against the control. The greatest decrease was observed after the use of 10 μg/dL Pb (2.36% reduction vs. control, *p* ≤ 0.005). Moreover, a significant decrease in the viability of cells treated with 10 μg/dL Pb was observed in comparison with 2.5 μg/dL Pb and 5 μg/dL Pb. A statistically significant difference was also observed between the cells cultured with Pb at 1.25 μg/dL and 5 μg/dL (Fig. 1).While evaluating the viability of THP-1 macrophages, statistically significant differences were found between all studied cell groups. The greatest decrease was observed from the use of 10 μg/dL Pb (5.29% reduction vs. control, *p* ≤ 0.005) (Fig. 2).

### The Effects of Lead on Early Apoptosis

**Fig. 3:**
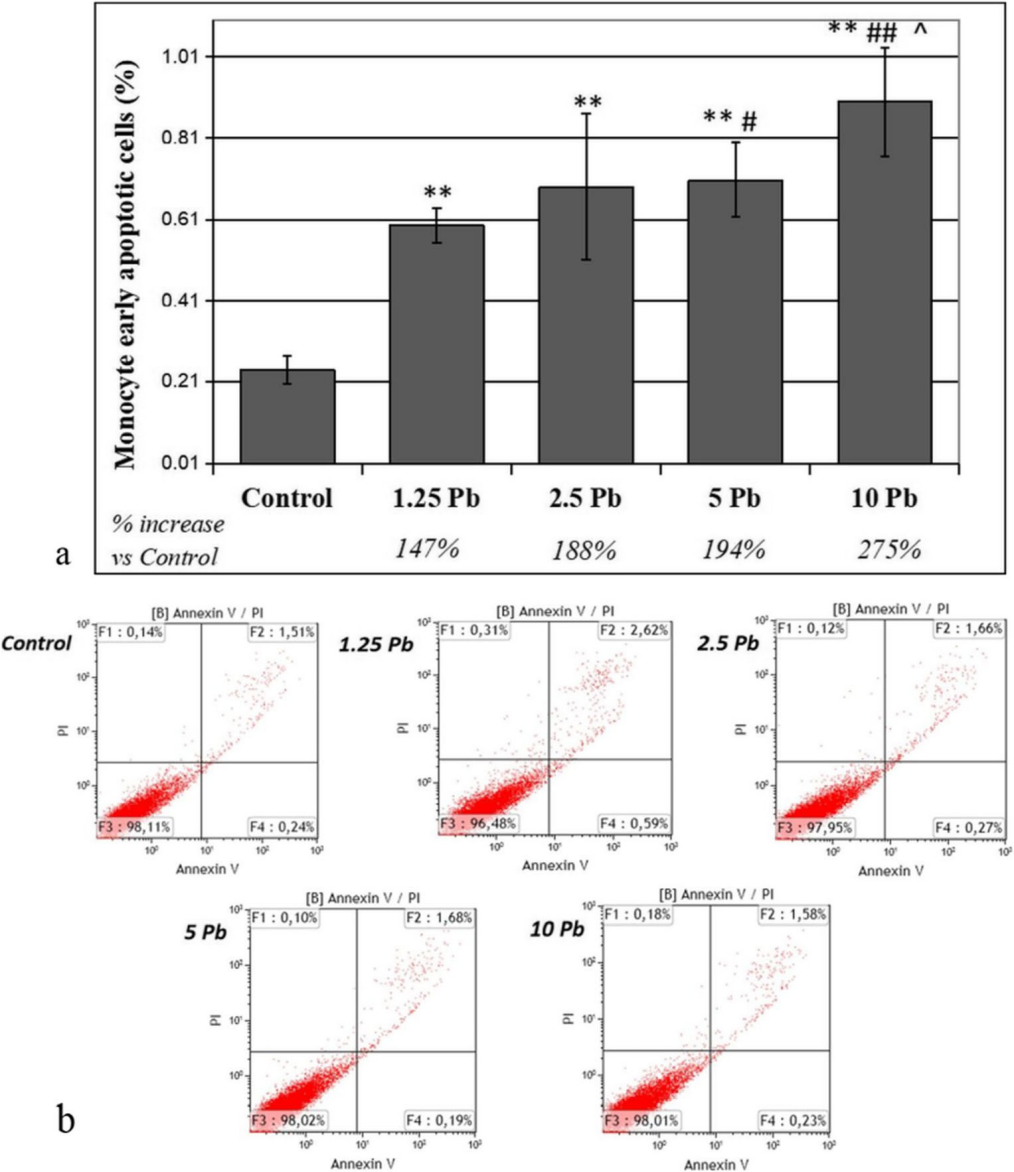
The effect of lead on early apoptosis in THP-1 monocytes cultured with various lead acetate solutions for 48 h. After incubation with PbAc, cells were further incubated with Annexin V-FITC (1 ng/mL) and propidium iodide (5 ng/mL) for 30 min in the dark and analyzed by flow cytometry. Results are expressed in percentage of apoptotic cells from *n* = 6 separate experiments (**a**). Lower left quadrant shows viable cells. Lower right quadrant, early apoptotic cells. Upper left quadrant, necrotic cells. Upper right quadrant, nonviable late apoptotic cells. Diagram of representative samples (**b**). **Statistically significant differences in comparison with the control (*p* ≤ 0.005). ##Statistically significant differences in comparison with 1.25 μg/dL Pb (*p* ≤ 0.005), #(*p* ≤ 0.05). ^Statistically significant differences in comparison with 5 μg/dL Pb (*p* ≤ 0.05). Control—cells incubated in RPMI medium with 10% FBS and without Pb

**Fig. 4:**
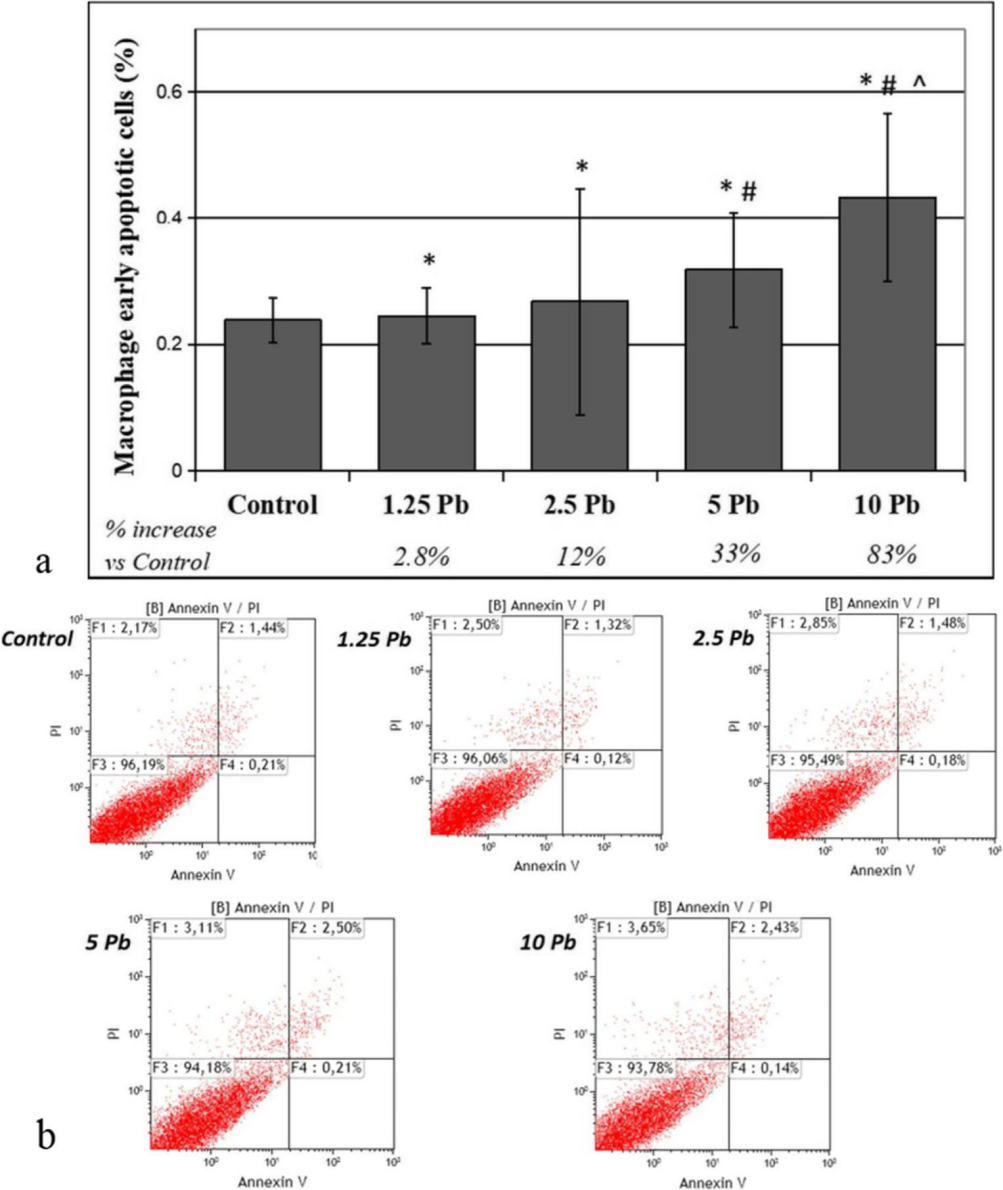
The effect of lead on early apoptosis in THP-1 macrophages cultured with various lead acetate solutions for 48 h. After incubation with PbAc, cells were further incubated with Annexin V-FITC (1 ng/mL) and propidium iodide (5 ng/mL) for 30 min in the dark and were analyzed by flow cytometry. Results are expressed in percentage of apoptotic cells from *n* = 6 separate experiments (**a**). Lower left quadrant shows viable cells. Lower right quadrant, early apoptotic cells. Upper left quadrant, necrotic cells. Upper right quadrant, nonviable late apoptotic cells. Diagram of representative samples (**b**). *Statistically significant differences in comparison with the control (*p* ≤ 0.05). #Statistically significant differences in comparison with 1.25 μg/dL Pb (*p* ≤ 0.05). ^Statistically significant differences in comparison with 2.5 μg/dL Pb (*p* ≤ 0.05). Control—cells incubated in RPMI medium with 10% FBS and without Pb

Using the Annexin V-FITC and PI double staining method, the degree of early apoptosis in THP-1 monocytes and macrophages was quantified (Figs. 3 and 4).

Forty-eight hours of incubation of THP-1 monocytes with Pb in the examined concentrations caused a significant dose-dependent increase in the percentage of early apoptotic cells (FITC+, PI−) of 147% (for 1.25 μg/dL) to 275% (for 10 μg/dL) compared with that in of the control (Fig. 3). The percentage of apoptotic cells after incubation with 5 μg/dL and 10 μg/dL was significantly greater than to 1.25 μg/dL. The percentages also differed significantly between the two highest concentrations (5 μg/dL and 10 μg/dL).

THP-1 macrophages exposed for 48 h to PbAc at each of the tested concentrations showed an increase in the percentage of early apoptotic cells compared with the control (from 2.8% for 1.25 μg/dL Pb to 83% for 10 μg/dL Pb). The percentage of early apoptotic cells after incubation in 5 μg/dL Pb and 10 μg/dL Pb also significantly increased compared with that in the lowest Pb concentration. The highest Pb concentrations also showed increased early apoptosis compared with 2.5 μg/dL Pb (Fig. 4).

### The Effects of Lead on Late Apoptosis/Necrosis

**Fig. 5:**
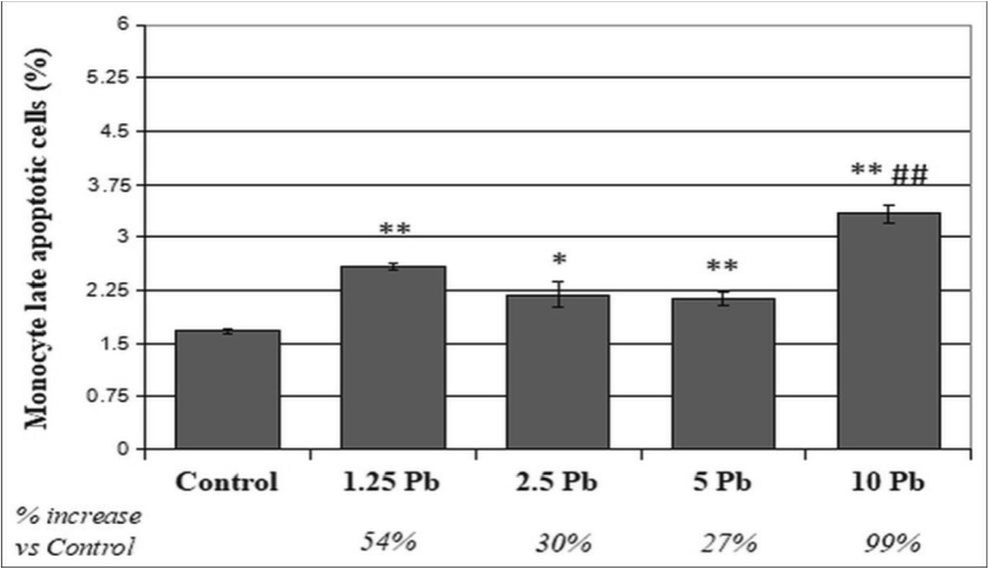
The effect of lead on late apoptosis/necrosis in monocytes cultured with various acetate solutions for 48 h. After incubation with PbAc, cells were harvested by scraping then were incubated with Annexin V-FITC (1 ng/mL) and propidium iodide (5 ng/mL) for 30 min in the dark and analyzed by flow cytometry. Results are expressed in percentage of apoptotic cells from *n* = 6 separate experiments. **Statistically significant differences in comparison with the control (*p* ≤ 0.005), *(*p* ≤ 0.05). ##Statistically significant differences in comparison with 5 μg/dL Pb (*p* ≤ 0.005). Control—cells incubated in RPMI medium with 10% FBS and without Pb

A 48-h incubation of THP-1 monocytes with PbAc caused a significant increase in the percentage of late apoptotic/necrotic cells (FITC +, PI +) at all tested PbAc concentrations compared with that of control, with the highest increase for 10 μg/dL Pb (99%). The difference was also statistically significant when comparing the cells exposed to the highest Pb concentrations, i.e., between 5 and 10 μg/dL Pb (Fig. 5).

**Fig. 6:**
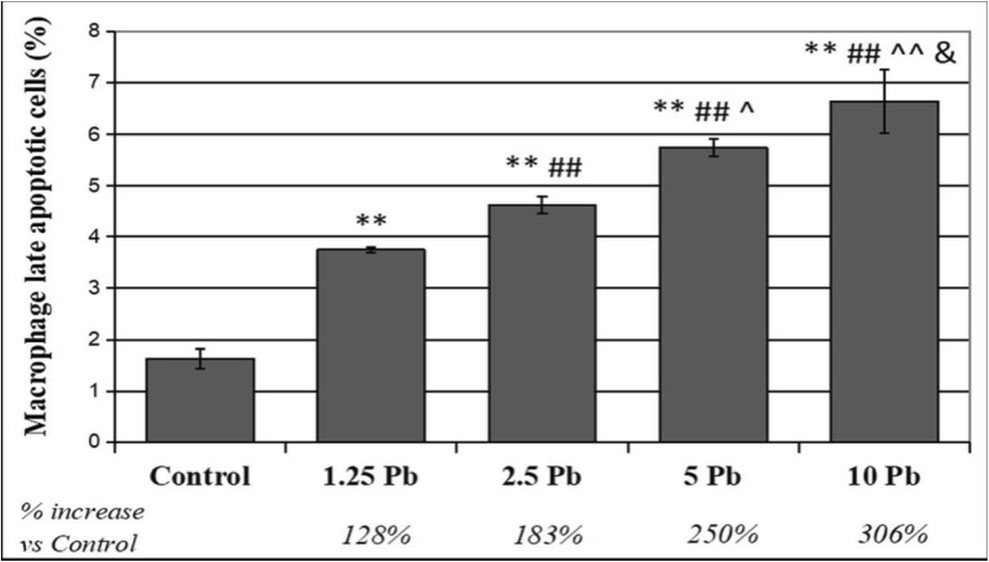
The effect of lead on late apoptosis/necrosis in macrophages cultured with various acetate solutions for 48 h. After incubation, cells were harvested by scraping, then incubated with Annexin V-FITC (1 ng/mL) and propidium iodide (5 ng/mL) for 30 min in the dark and analyzed by flow cytometry. Results are expressed in percentage of apoptotic cells from *n* = 6 separate experiments. **Statistically significant differences in comparison with the control (*p* ≤ 0.005). ##Statistically significant differences in comparison with 1.25 μg/dL Pb (*p* ≤ 0.005). ^^Statistically significant differences in comparison with 2.5 μg/dL Pb (*p* ≤ 0.005), ^(*p* ≤ 0.05). Statistically significant differences in comparison with 5 μg/dL Pb (*p* ≤ 0.05). Control—cells incubated in RPMI medium with 10% FBS and without Pb

THP-1 macrophages showed statistically significant differences between each of the applied Pb concentrations. The highest increase in the percentage of apoptotic cells in relation to control was observed after exposure to 10 μg/dL Pb (306% of control) (Fig. 6).

### The Concentration of Lead in THP-1 Macrophages

**Fig. 7:**
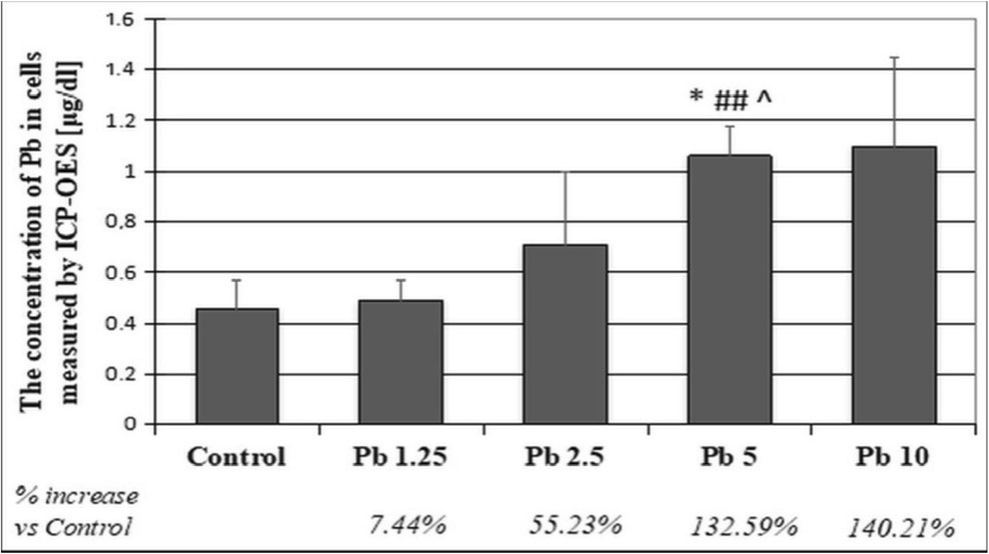
The concentration of lead in THP-1 macrophages after incubation with various acetate solutions. THP-1 macrophages were cultured with lead acetate solutions for 24 h. After incubation, cells were harvested by scraping and subjected to microwave mineralization using MARS 5 system, CEM. Ion accumulation was measured by ICP-OES. The values were expressed as an arithmetic mean (± SD) of *n* = 6 measurements for each concentration. *Statistically significant differences in comparison with the control (*p* ≤ 0.05). ##Statistically significant differences in comparison with 1.25 μg/dL Pb (*p* ≤ 0.005). ^Statistically significant differences in comparison with 2.5 μg/dL Pb (*p* ≤ 0.05). Control—cells incubated in RPMI medium with 10% FBS and without Pb

**Fig. 8:**
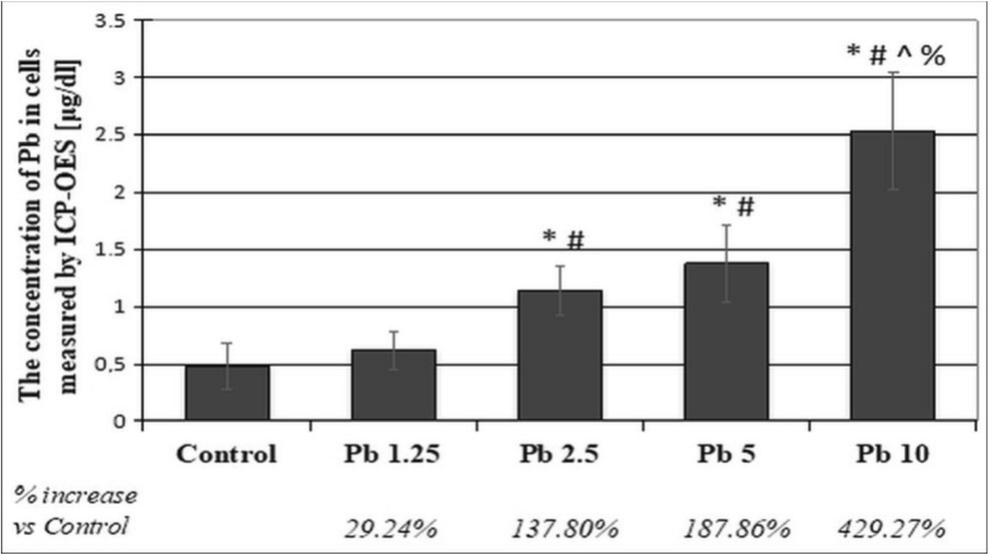
The concentration of lead in THP-1 macrophages after incubation with various acetate solutions. THP-1 macrophages were cultured with lead acetate solutions for 48 h. After incubation, cells were harvested by scraping and subjected to microwave mineralization using MARS 5 system, CEM. Ion accumulation was measured by ICP-OES. The values were expressed as an arithmetic mean (± SD) of *n* = 6 measurements for each concentration. *Statistically significant differences in comparison with the control (*p* ≤ 0.05). #Statistically significant differences in comparison with 1.25 μg/dL Pb (*p* ≤ 0.05). ^Statistically significant differences in comparison with 2.5 μg/dL Pb (*p* ≤ 0.05). %Statistically significant differences in comparison with 5 μg/dL Pb (*p* ≤ 0.05). Control—cells incubated in RPMI medium with 10% FBS and without Pb

**Fig. 9:**
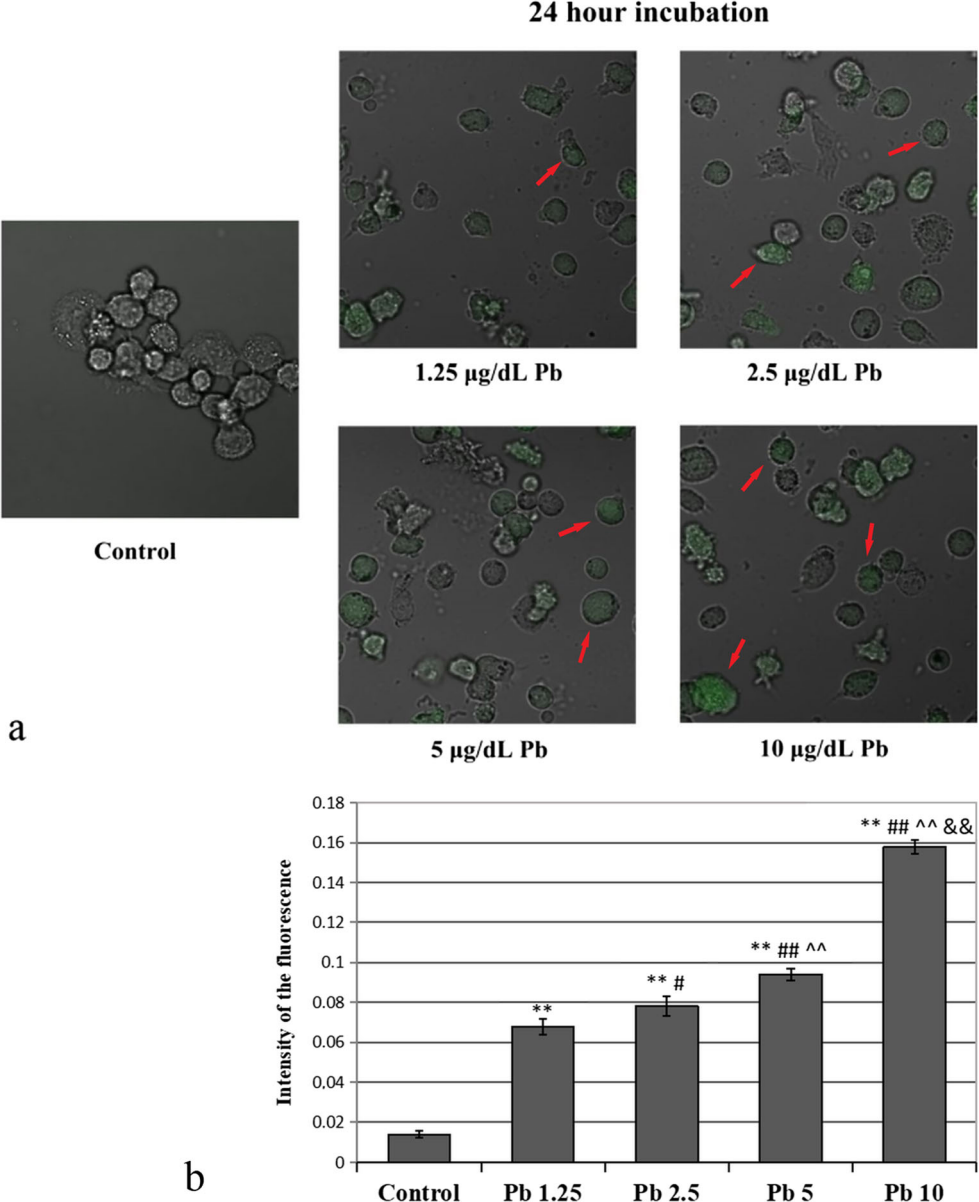

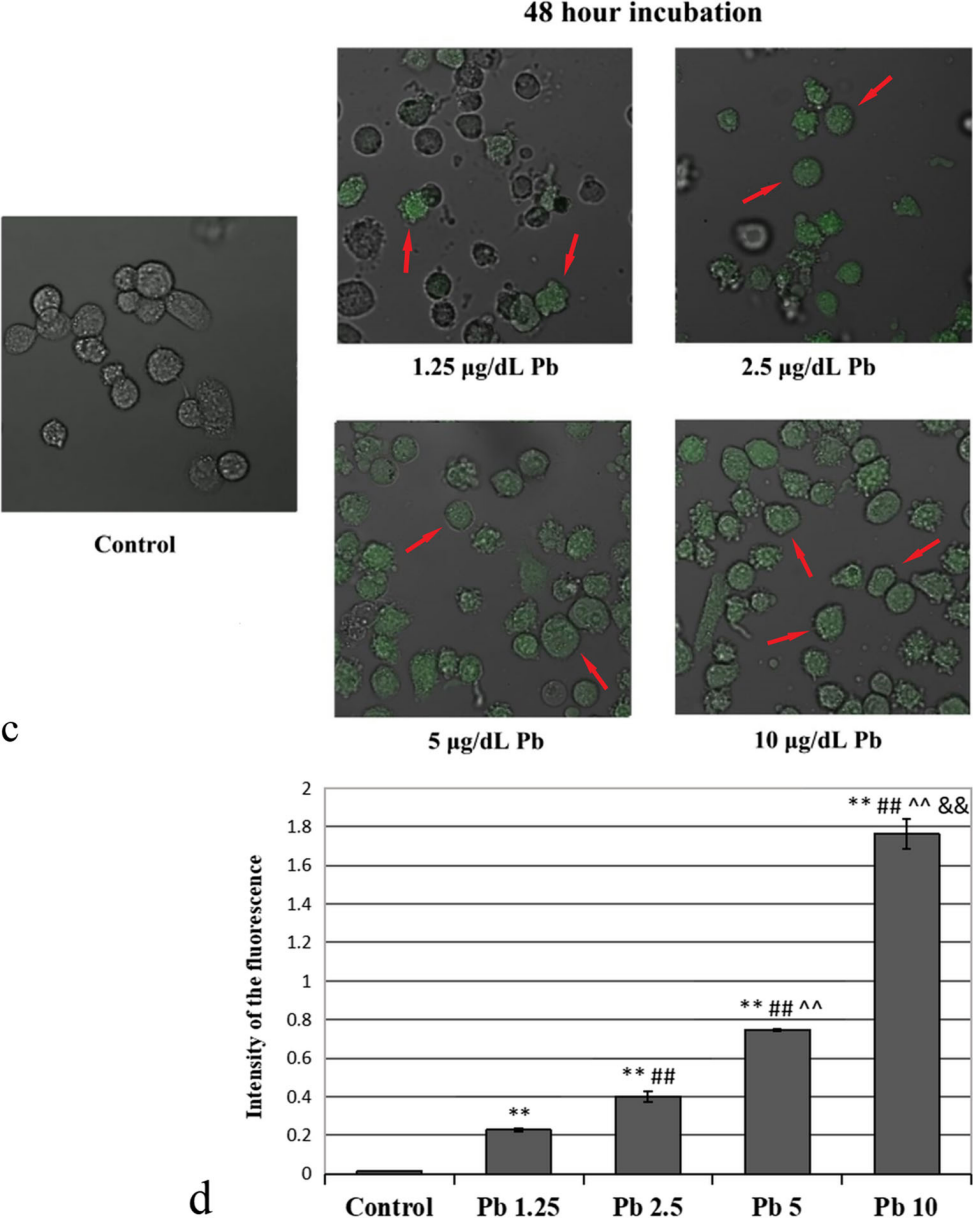
Confocal microscope images of cultured THP-1 macrophages incubated for 24 h (**a**) or 48 h (**c**) with Leadmium™ Green AM dye from control and Pb-treated cells; quantitative measurements of the fluorescence intensity after 24-h (**b**) and 48-h incubation (**d**). Quantitative measurements of the fluorescence intensity performed by an Asys UVM 340 plate reader, normalized to the number of cells on the plate. The intensity of red-green fluorescence in the cultured with Pb macrophages was significantly higher with respect to control macrophages. Experiments were repeated six times with similar results; therefore, the presented pictures may be deemed representative. The values were expressed as an arithmetic mean (± SD) of *n* = 6 measurements for each concentration. **Statistically significant differences in comparison with control (*p* ≤ 0.005). ##Statistically significant differences in comparison with 1.25 μg/dL Pb (*p* ≤ 0.005), #(*p* ≤ 0.05). ^^Statistically significant differences in comparison with 2.5 μg/dL Pb (*p* ≤ 0.005). &&Statistically significant differences in comparison with 5 μg/dL Pb (*p* ≤ 0.005). Control—cells incubated in RPMI medium with 10% FBS and without Pb

Our results indicate that macrophages were more sensitive to Pb than monocytes. Exposed to the same concentrations, they showed lower viability and a higher percentage of necrotic cells. Therefore, the assessment of the degree of accumulation of Pb was performed on THP-1 macrophages. We applied two incubation times: 24 and 48 h (Figs. 7, 8, and 9).

Longer exposure to Pb resulted in a higher Pb accumulation in the cells studied. Both times lead to a significant increase in the accumulation depending on the dose used. Cell evaluation after 24 h showed the highest Pb accumulation after exposure to the highest Pb level (10 μg/dL). Statistically significant differences were observed between the cells cultured with Pb at a concentration of 5 μg/dL and those exposed to 1.25 μg/dL and 2.5 μg/dL Pb, vs. control group (Figs. 7 and 9).

A 48-h incubation resulted in increased accumulation of Pb in cells exposed to 10 μg/dL Pb compared with that in the control, 1.25-μg/dL cells, and 5-μg/dL cells. The increase in the amount of Pb in cells exposed to 5 μg/dL Pb and 2.5 μg/dL Pb was significant in comparison with that in 1.25 μg/dL Pb and control cultures (Figs. 8 and 9).

## Discussion

In our experiment, we investigated the influence of lead on apoptosis and necrosis in THP-1 monocytes and macrophages. We also examined the ability of macrophages to accumulate Pb, depending on the length of exposure and various levels of Pb concentrations reflecting environmental exposure in urban areas [8]. As a result of incubation with Pb, THP-1 cells, both monocytes and macrophages, showed a concentration-dependent reduction in viability and an increase in the percentage of early and late apoptotic cells.

Our results are in agreement with the results obtained by other research groups investigating various cell lines and in vivo animal models, showing Pb causing apoptosis of neurons [21, 29, 30, 43], such as in hippocampal neurons [30, 52] and mouse hippocampal neuronal cell line HT-22 [34], retinal cells [31, 53], and human leukemia cells (HL-60) [32, 33]. Only peripheral blood mononuclear cells (MNC) showed no apoptosis even at 500 μM Pb [54]. The concentrations used by other researchers were many times higher than in this study [32–34], yet our results showed a significant decrease in viability even after exposure to the very low concentration of 1.25 μg/dL Pb.

The demonstrated sensitivity to much lower Pb concentrations in our study may be due to the use of different research models and incubation times. Karri et al. hypothesized that the duration of exposure is a key factor influencing the occurrence of toxic or lethal Pb effect on the cells studied. They tested three exposure periods: acute (1 day), subchronic (3 days), and chronic (8 days) assays. They chose hippocampal cell line HT-22 as the research model, and 10 different Pb concentrations ranging from 10 to 100 μM Pb. A clear, time- and concentration-dependent cytotoxic effect of Pb on the examined cells was observed [34].

The mechanism of Pb entry into the target cells is still not well understood [55]. Thanks to its ability to mimic other elements, Pb most likely uses the normal function of protein transporters. By replacing physiologically essential cations (e.g., Ca^2+^), it can enter the cell interior through passive and active transport processes [56]. The basic mechanism of active transport, i.e., the calcium pump which uses ATP hydrolysis to transport calcium into cells, is considered specific for Ca^2+^ ions, but there is some evidence that several other bivalent cations can replace them, including Pb^2+^ ions [57, 58]. Passive transport of Pb, thanks to which Pb may pass through the cell membrane in any direction, is stimulated by bicarbonates (HCO_3_^−^) and does not depend on the external concentrations of Na^+^, K^+^, or Ca^2+^ [59].

An in vivo study on a rat model suggested that pH-dependent passive Pb transport in the form of monovalent permeating species of the type PbOH^+^ is the mechanism behind the breach of the blood-brain barrier by Pb [60], resulting in Pb accumulation in different parts of the brain tissue [61]. Under physiological conditions, macrophages formed from monocytes become long-lived cells, develop specialized functions, and are therefore more resistant to constitutive apoptosis than monocytes [62]. Our results, however, suggest that macrophages are more sensitive to Pb than monocytes, as exposure to the same concentrations resulted in a lower viability and higher percentage of necrotic cells. It is possible that Pb significantly interfered with macrophage functions and thus, the degenerated cells more readily entered the apoptotic pathway.

Pb accumulation by macrophages can have serious cytotoxic and pro-inflammatory consequences [11]. For example, it may increase lipid peroxidation in macrophages [63–70], with the accumulation of oxidized lipid leading to the formation of foam cells and the development of inflammation [37, 71–73].

Chronic exposure to Pb also results in elevated concentrations of total cholesterol and triglycerides [74, 75], with increased concentrations of fatty acids in blood and macrophages being an important factor in the development of atherosclerosis [76]. Baranowska-Bosiacka et al. showed that environmental Pb concentrations may also be a risk factor affecting fatty acid concentrations, inducing oxidative stress and increasing malondialdehyde (MDA) concentration in macrophages, leading to foam cell formation and development of inflammation [77].

The toxicity of Pb can also be associated with its influence on the activity of antioxidant enzymes. A study [78] evaluated the level of oxidative stress in rat brains exposed pre- and neonatal to Pb, expression of mRNAs, proteins, and activity of the most important antioxidant enzymes (copper/zinc superoxide dismutase (SOD1), manganese superoxide dismutase (SOD2), glutathione peroxidase (GPx), phospholipid hydroperoxide glutathione peroxidase (GPx4), catalase (CAT), glutathione reductase (GSR), and glutathione (GSH)). Despite the low level of Pb in the blood (10 μg/dL), a decrease in the activity of some enzymes was observed, as well as in their mRNA and protein expression, associated with an increase in MDA and CAT expression, especially in the hippocampus. The same paper also suggested that a disturbed oxidant-antioxidant balance in both neurons and glia may be a potential mechanism underlying the observed adverse effects of Pb [78].

The apoptosis of macrophages observed in our experiment may occur through the activation of many different pathways, e.g., through the Fas pathway and mitochondrial pathways. FasL is activated on the surface of cells overloaded with free cholesterol [79] and an increase in Bax levels and a release of cytochrome c result in the activation of caspase-9 and other effector caspases [80]. The accumulation of free cholesterol in the endoplasmic reticulum (ER) of cells also causes the activation of unfolded protein response (UPR) and C/EBP homologous protein (CHOP)–induced apoptosis via p38 mitogen-activated protein kinases (MAPK). Devries-Seimon et al. believe that this pathway requires the action of scavenger receptor class A (SRA) and the c-Jun NH2-terminal kinase pathway [81].

Heavy metals other than Pb can also initiate macrophage apoptosis. For example, macrophages exposed to beryllium produce an increased amount of reactive oxygen species (ROS) [82] and undergo apoptosis due to the activation of caspases [83]. Macrophages exposed to mercury die as a result of the induction of apoptosis and necrosis through different activation paths. Exposure to Hg increases the intracellular concentration of Ca^2+^ increases, leading to increased production of ROS which then activates p38, responsible for elevated apoptosis and necrosis of cells through its influence on caspase-3 and expression of tumor necrosis factor α (TNF-α) [84]. A study on cadmium-incubated macrophages has shown apoptosis induced via mitochondrial pathways, with a decrease in membrane potential of mitochondria and an increase in ROS production [49]. Cadmium can also activate extracellular signal-regulated kinase (ERK) [85] and JNK MAPKs [86, 87] and increases the expression of mRNA Bax while decreasing the expression of Bcl-2 (increase in Bax/Bcl-2 ratio) [87–91]. Olszowski et al. showed that these mechanisms can be induced by cadmium event at nanomolar concentrations [49].

Lead-induced apoptosis also seems to depend on mitochondria, as Pb is able to affect mitochondrial calcium homeostasis. Both calcium and lead depolarize mitochondrial membranes by opening permeability transition pores (PTP) [53]. Pb affects the levels of intercellular oxidants [92], adenylate energy charge value (AEC), and ATP production [24, 93, 94] and increases ROS production, all of which—through constant demand for antioxidants—lead to a depletion of resources [95]. This results in the destabilization of calcium homeostasis via disrupted electron transport, decreased ATP concentration, and membrane ion channel disruption [96], ultimately leading to cell apoptosis. Increased ROS concentration results in a disturbance of the cellular lipid bi-layer and thus disturbs the cellular transport of Ca^2+^. Compromised cellular membranes are indicated by a loss in mitochondrial membrane potential (MMP), resulting in disturbances in the balance of such molecules as Bcl-2 and Bax [97]. The changed Bcl-2/Bax ratio causes a release of cytochrome c and activation of caspases leading to apoptosis [98, 99]. Finally, research on the brains of rats with long-term exposure to Pb showed a significant increase in ROS, neuronal synthase of nitric oxide, and intracellular calcium levels; a decrease in membrane potential; a cytochrome c release; and changes in the Bcl-2/Bax ratio, which confirm mitochondrial-dependent Pb-induced apoptosis [100].

In conclusion, despite the use of low Pb concentrations in our experiment, both monocytes and macrophages showed both a concentration and time-dependent decrease in viability, accompanied by a simultaneous increase in the percentage of early and late apoptotic cells. Macrophages seemed to react more strongly to Pb and therefore were selected to evaluate the Pb accumulation after 24 and 48 h of incubation. Longer exposure times resulted in a stronger accumulation, with a similar dependence on concentration. The results indicate that environmental exposure to low concentrations of Pb compounds may significantly impair the function of macrophages. The resulting increase in the number of apoptotic cells may contribute to the development of many pathologies in the entire organism. Our research indicates that blood lead levels as low as 1.25 g/dL can cause apoptotic changes in monocytes and macrophages. This suggests that the safety threshold Pb levels should even lower. In addition, we should try to further curb environmental exposure to Pb, as well as continue intense research on the mechanisms of Pb toxicity.
